# Targeting Clic1 for the treatment of obesity: A novel therapeutic strategy to reduce food intake and body weight

**DOI:** 10.1016/j.molmet.2023.101794

**Published:** 2023-08-20

**Authors:** Rizaldy C. Zapata, Dinghong Zhang, Dongmin Yoon, Chanond A. Nasamran, Daisy R. Chilin-Fuentes, Avraham Libster, Besma S. Chaudry, Mariela Lopez-Valencia, Devasena Ponnalagu, Harpreet Singh, Michael Petrascheck, Olivia Osborn

**Affiliations:** 1 Division of Endocrinology and Metabolism, School of Medicine, University of California San Diego, La Jolla, CA, 92093, USA; 2 Center for Computational Biology & Bioinformatics, Department of Medicine, University of California, San Diego, La Jolla, CA, USA; 3 Department of Physiology and Cell Biology, The Ohio State University, Columbus, OH, USA; 4 Dorothy M. Davis Heart and Lung Research Institute, The Ohio State University, Columbus, OH, USA; 5 Department of Molecular Medicine, The Scripps Research Institute, 10550 North Torrey Pines Road, La Jolla, CA, 92037, USA; 6 Department of Neuroscience, The Scripps Research Institute, 10550 North Torrey Pines Road, La Jolla, CA, 92037, USA

**Keywords:** Obesity, Food intake, Clic1, Hyperphagia, Body weight regulation

## Abstract

**Objective:**

Despite great advances in obesity therapeutics in recent years, there is still a need to identify additional therapeutic targets for the treatment of this disease. We previously discovered a signature of genes, including Chloride intracellular channel 1 (Clic1), whose expression was associated with drug-induced weight gain, and in these studies, we assess the effect of Clic1 inhibition on food intake and body weight in mice.

**Methods:**

We studied the impact of Clic1 inhibition in mouse models of binge-eating, diet-induced obese mice and genetic models of obesity (Magel2 KO mice).

**Results:**

Clic1 knockout (KO) mice ate significantly less and had a lower body weight than WT littermates when either fed chow or high fat diet. Furthermore, pharmacological inhibition of Clic1 in diet-induced obese mice resulted in suppression of food intake and promoted highly efficacious weight loss. Clic1 inhibition also reduced food intake in binge-eating models and hyperphagic Magel2 KO mice. We observed that chronic obesity resulted in a significant change in subcellular localization of Clic1 with an increased ratio of Clic1 in the membrane in the obese state. These observations provide a novel therapeutic strategy to block Clic1 translocation as a potential mechanism to reduce food intake and lower body weight.

**Conclusions:**

These studies attribute a novel role of Clic1 as a driver of food intake and overconsumption. In summary, we have identified hypothalamic expression of *Clic1* plays a key role in food intake, providing a novel therapeutic target to treat overconsumption that is the root cause of modern obesity.

## Highlights

• Clic1 is expressed in the hypothalamus.
• Clic1 knockout mice eat less and weigh less than littermate controls.
• Clic1 inhibition reduces food intake and induces weight loss in obese mice.

## 1 Introduction

Modern-day human eating behavior is characterized by continuous availability and overconsumption of energy-rich food which are key drivers of the obesity epidemic. Over one-third of US adults are obese [1] causing a formidable socioeconomic challenge and widely impacting public health [2]. Despite great advances in efficacious pharmacotherapy for obesity in recent years, there is still a general lack of understanding of the basic biological mechanisms that contribute to obesity and a need to identify additional therapeutic targets [3]. Using a multispecies approach, originating with a screen in *Caenorhabditis elegans*, we previously discovered a signature of genes, including Chloride intracellular channel 1 (Clic1), whose expression was associated with drug-induced weight gain [4]. Interestingly, antipsychotic drugs drive a rapid change in food intake, in both rodents [[4], [5], [6], [7]] and humans [8,9], but the underlying pathways driving this side effect are largely unknown [[10], [11], [12], [13]]. We hypothesized that pathways contributing to hyperphagia and weight gain could be targeted in the reverse direction to stimulate reduction in food intake and drive weight loss. Clic1, a 241 amino acid protein, exhibits a dual nature, existing in two distinct forms: a soluble enzymatic form and a membrane-associated ion channel form [14]. The reversible transformation from the soluble, predominant glutathione-S transferase (GST)-like structure [15] to that of an integral membrane protein form [16] is prompted by various stimuli including alterations in cellular pH and oxidative stress [[17], [18], [19]]. This translocation phenomenon has been observed in diverse cell types, such as endothelial cells [20,21] and microglia [22,23]. Importantly, the Clic1 inhibitor, IAA94, functionally inhibits Clic1 by preventing translocation to the transmembrane region, and Clic1 remains predominantly in the cytoplasmic compartment [21,24]. Therefore, a crucial aspect of Clic1's function lies in the subcellular localization [25]. Previous studies have identified that whole body Clic1 KO mice, are leaner than WT controls in their early development (age 3–11 weeks of age) [26]. In these studies, we investigate the role of Clic1 in food intake, weight gain and the investigate the impact of Clic1 inhibition as a potential treatment for obesity and metabolic health.

## 2 Methods

### 2.1 In vitro studies in hypothalamic cells

Adult mouse hypothalamic cell line (mHypoA-59, CLU468 cells, Cedarlane) were cultured as previously described. Briefly, cells were grown and maintained in high-glucose, pyruvate-free DMEM supplemented with 10% fetal bovine serum, l-glutamine (Cat. 25030081, Gibco, NY), and 10 u/ml of penicillin and 10 μg/ml of streptomycin (Cat. 15149-122, Gibco) of in a 5% CO_2_ environment. Cells were ‘fasted’ for 8 h using the same growth media but devoid of fetal bovine serum, with or without IAA94 (40 μM).

### 2.2 Gene expression

RNA was extracted, converted to cDNA and relative expression analyzed by qPCR. Gene expression was calculated after normalization to the housekeeping gene *Hprt1 and Pgk1* using the Δ^ΔCt^ method. Gene expression was calculated relative to experimental controls. Primer sequences used to measure gene expression are detailed in Supplemental Table 1.

### 2.3 Clic1 localization studies

Cytoplasmic and membrane-bound hypothalamic proteins from lean and obese mice fed with 60% high-fat diet for 8 weeks were isolated using ProteoExtract Native Protein kit (444810, Calbiochem) as recommended by the manufacturer. Proteins (20 μg) were fractionated in 4–15% Mini PROTEAN TGX acrylamide gels, transferred to PVDF, blocked with 5% BSA, incubated with the primary antibody overnight and secondary antibody for 60 min before detection using ECL. Band intensities where quantified using densitometer in ImageLab. The following antibodies were used: anti-Clic1 (1:100, sc-81873, Santa Cruz Biotechnology), anti-beta actin (1:2000, 3700, Cell Signaling), anti-Na^+^, K^+^-ATPase (1:1000, 3010, Cell Signaling), anti-mouse IgG (1:4000, 115035003, Jackson Immunoresearch), anti-rabbit IgG (1:4000, NA934V, GE Healthcare).

### 2.4 Acute fasting and re-feeding studies

All mice studies were approved by UCSD IACUC. WT C57BL/6 mice (stock #000664) were purchased from Jackson labs at 9 weeks of age. At 12 weeks of age, mice were fasted for 23 h and then re-fed for one hour before sacrificed. Hypothalami were dissected, frozen and later analyzed for gene expression studies.

### 2.5 Clic1 KO mice studies

We obtained the whole body Clic1 knockout (KO) mice, on a CD-1 background, from Prof. John C. Edwards, St Louis University [26]. KO mice and respective WT littermate controls were fed normal chow. The food novelty test was conducted in a similar way as previously described [27]. In brief, mice have a natural phobia to novelty, therefore, when WT mice are exposed to a novel pellet of highly palatable food, they have to overcome this phobia to consume the novel food and this partially models their drive, (or lack of motivation) to consume palatable food. During the 3-day experiment, food was withdrawn from 9-week chow fed mice 2 h before the onset of the dark period. A pellet of 45% high fat diet, HFD (D12451, Research Diets) was introduced to the food hoppers at the onset of the dark period and were removed and replaced with chow diet after 20 min. Glucose tolerance test was conducted as described previously [28,29]. In brief, mice were fasted for 6 h, injected IP with glucose (1 g/kg, Hospira, Lake Forest, IL) and blood drawn at 0, 10, 30, 60, 90, and 120 min after the injection for blood glucose determination using an Easy Step blood glucose monitor (Home Aid Diagnostics Inc, FL). Plasma insulin was measured in the fasted and 10 min blood sample using 900-MPMI-02 (Alpco, NH).

In a second cohort, male WT and Clic1 KO mice were acclimated for 3 days in metabolic chambers (Promethion, Sable Systems, NV). Food intake, body weight, respiratory gas exchange and activity were measured in the next 7 days. Data was organized using ExpeData macros 1 and 13. Energy expenditure was normalized by body weight.

Indanyloxyacetic acid-94 (IAA94, HY-12693, Medchem Express) is a chloride intracellular channel blocker. To assess whether IAA94s hypophagic property is mediated though its inhibitory action against Clic1, we administered IAA94 to chow-fed WT and KO mice at 12 weeks of age by intraperitoneal (IP) injection, at a dose of 10 mg/kg, and food intake measured over the next 24 h.

### 2.6 Binge eating studies

The experiment was conducted as previously described [30]. WT mice were divided into one of 4 experimental groups; ‘Continuous’ (C-Veh or C-IAA94) and ‘intermittent’ (I-Veh or I-IAA94), (n = 5/group) ‘Continuous’ groups had *ad libitum* access to high fat diet (HFD) (DS12492, 60% calories from fat) throughout the study. The ‘intermittent’ groups received an initial 48 h acclimation to HFD after which the HFD was removed for 5 days and only the NC was available *ad libitum*. The HFD was then presented back to the mice for a short 2.5-h period and food intake measured.

### 2.7 RNA sequencing

RNA from the hypothalamus of WT and KO mice was isolated using RNeasy Lipid tissue kit (Qiagen, 74804) according to the manufacturer's recommended protocol. RNA sequencing was conducted at UCSD Genomic Core. RNA quality and concentration were evaluated using Tapestation and samples with a RIN score of more than 8.0 underwent library preparation and RNA sequencing using Novaseq S4. Quality control of the raw fastq files was performed using the software tool FastQC v0.11.8. [31]. Sequencing reads were trimmed with Trimmomatic v0.38 [32] and aligned to the mouse genome (GRCm38p6 [33]) using the STAR aligner v2.5.1a [34]. Read quantification was performed with RSEM [35] v1.3.0 and the Ensembl release 98 annotation [36]. The R BioConductor packages edgeR [37] and limma [38] were used to implement the limma-voom9 method for differential expression analysis. In brief, lowly expressed genes—those not having counts per million (cpm) ≥ 1 in at least 5 of the samples—were filtered out and then trimmed mean of M-values (TMM) [39] normalization was applied. The experimental design was modeled upon condition (∼0 + condition). The voom method was employed to model the mean–variance relationship in the log-cpm values, after which lmFit was used to fit per-gene linear models and empirical Bayes moderation was applied with the eBayes function. Significance was defined by using an adjusted *p*-value cut-off of 0.05 after multiple testing correction [40] using a moderated t-statistic in limma. Functional enrichment of the differentially expressed genes was performed using WebGestalt [41] (including GSEA [42]), GSVA [43], SPIA [44], and fgsea [45].

### 2.8 Investigation of dose dependent effect of IAA94 and comparison with Liraglutide treatment alone, or combination of Liraglutide and IAA94 on weight loss

Male C57BL6 mice, age 10 weeks, were fed 60% HFD (DS12492, Research Diets) for 8 weeks. At 18 weeks of age, mice were randomly divided into five groups, (1) Vehicle (n = 9), (2) Low dose IAA94 (n = 7, 10 mg/kg, MedChem Express, HY-12693), (3) High dose IAA94 (n = 8, 50 mg/kg), (4) Liraglutide (n = 6, 0.4 mg/kg [46,47], SelleckChem, NN2211) and (5) Liraglutide + High dose IAA94 (n = 5). All drugs were resuspended in vehicle (Saline + 4% DMSO, 10% Tween 80). At day 8, mice were placed in metabolic chambers (Promethion, Sable Systems, NV) in two staggered cohorts where daily food intake, body weight, oxygen consumption, carbon dioxide production, energy expenditure, activity and respiratory exchange ratio (RQ) were measured at 5 min intervals throughout the daily light and dark cycle, for 7 days. Fat mass and lean mass were determined by Echo MRI analysis at the end of the study. Plasma levels of ALT (EBC-K235, Elabscience) were determined according to manufacturers' instructions.

### 2.9 Magel2 KO mice studies

Magel2 KO mice recapitulates some aspects of Prader–Willi syndrome. Male Magel2 KO mice and wildtype littermates were provided courtesy of Dr. Marcelo Dietrich, Yale University. After 7 days of acclimation, mice were injected daily with either vehicle or IAA94 (IP) for 9 days. Food intake, weight gain and gonadal adipose tissue weights were measured.

### 2.10 Psychiatric behavioral analysis

Marble burying is performed as previously described to assess compulsive behavior. Standard polycarbonate rat cages were used with fresh bedding for every mouse. Glass toy marbles were placed on the surface of the bedding in a 4 across and 5 down pattern. The mouse was placed into a corner of the cage and covered with the filter-top lid on the cage. The mouse was allowed to remain undisturbed in the cage for 30 min. The marble is considered buried if two-thirds of its surface is covered with bedding.

Forced swim test was conducted using published protocols to evaluate depression-like behavior. 5L glass beaker was filled with tap water set at room temperature up to the 3.5L mark. The videorecorder was started before the mouse was gently lowered in to the water. The mouse was allowed to swim for 6 min. The mouse was then dried and returned to its home cage. A total of 4 mice were studied in each session, with beakers separated by black dividers. Three independent investigators evaluated the time the mice were mobile and immobile during last 4 min of the test. Mobility is defined as any movements other than those necessary to balance the body and keep the head above the water. Increased immobility is an indication of depression-like behavior.

Elevated plus maze was conducted using published protocols to assess anxiety-like behavior. The apparatus used was in a + configuration which comprised of two open arms (25 × 5 cm) across from each other and perpendicular to two closed arms (25 × 5 cm) with a center platform (5 × 5 cm). The closed arm was equipped with 10 cm walls. The apparatus stood 50 cm above the floor. The test was conducted in a room with 100 lux light. The mouse was placed in the center area and was allowed to roam around the maze for 5 min. The experiment was recorded using a video camera which was analyzed by 3 independent investigators for number of exits to and the time spent in the open field.

## 3 Results

### 3.1 Fasting induces increased expression of Clic1 in the arcuate nucleus

**Figure 1:**
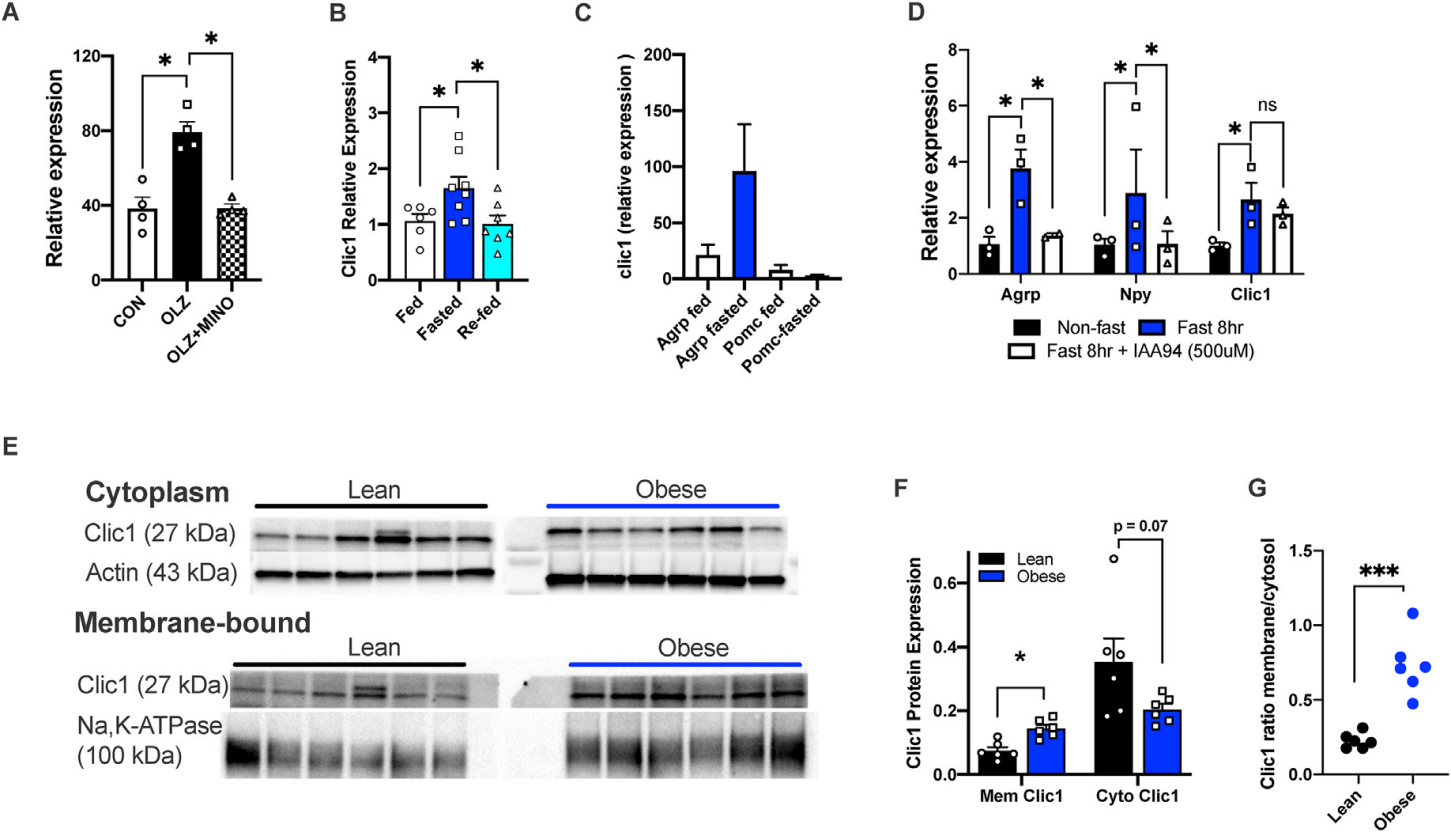
**Hypothalamic Expression of *Clic1*.A.** Hypothalamic expression of *clic1* in control, OLZ and OLZ + minocycline (MINO) treated mice (n = 4/group) determined from RNA seq studies (GSE119772) [4]. **B**. Hypothalamic expression of *Clic1* in *ad libitum* fed, 24 h fasted and 23 h fasted mice and then re-fed for 1 h (n = 6–8 per group) determined by quantitative PCR. **C**. Expression of *Clic1* in Agrp and Pomc neurons, using RNA seq data from Henry et al., 2015, (GSE93374) [48]. **D.***Clic1*, *Npy* and *Agrp* expression in non-fasted, 8-h fasted, and 8-h fasted + IAA94 treated hypothalamic cells (HypoA-59, n = 3/group). **E–F.** Immunoblots and densinometric quantification of Clic1 membrane (Mem) and cytosolic (Cyto) localization in the hypothalamus of lean and obese mice (n = 6/group) and **G.** ratio of membrane and cytosolic Clic1. A, B, D, ∗*p* < 0.05 One-way ANOVA followed by Two-stage linear step-up procedure of Benjamini, Krieger and Yekutieli with 0.05 FDR. F. ∗*p* < 0.05 Two-way ANOVA followed by Two-stage linear step-up procedure of Benjamini, Krieger and Yekutieli with 0.05 FDR. G. ∗∗∗*p* < 0.01 Student's t-test.

We recently identified a gene signature that was specifically associated with antipsychotic-induced hyperphagia which includes *Clic1* [4]. Hypothalamic expression of *Clic1* was significantly elevated in olanzapine-treated mice, and expression reduced by co-treatment with an adjuvant drug, minocycline, that lowered food intake (Figure 1A). To determine if hypothalamic expression of *Clic1* had a broader role in food intake, beyond antipsychotic-induced hyperphagia, we then measured its expression in response to fasting and re-feeding in mice. We found *Clic1* expression was significantly elevated in response to fasting and returned to basal levels one hour after re-feeding (Figure 1B). We then used cell type specific transcriptomics data [48] to determine expression levels of *Clic1* in two key neuronal populations in the arcuate nucleus (ARC) that play a key role in food intake. Notably, the ARC senses and integrates peripheral energy signals [49], such as blood glucose concentration, ghrelin, leptin and insulin [50]. *Clic1* is expressed at a low level in both orexigenic Neuropeptide Y (Npy)/Agouti-related peptide (Agrp) and anorexigenic proopiomelanocortin (Pomc) neurons but is potently and specifically elevated in Npy/Agrp neurons in response to fasting (Figure 1C).

### 3.2 Obesity results in an increase proportion of hypothalamic Clic1 localized to the membrane

Clic1 is a particularly unusual protein as it exists in both soluble (enzymatic form) and membrane-associated (ion channel) forms, thus, localization is a key element of Clic1 function [25]. Importantly, in endothelial cells [21] and cancer models [51], Clic1 inhibitor, Indanyloxyacetic acid (IAA) has been shown to functionally inhibit Clic1 by preventing translocation to the transmembrane region, and Clic1 remains predominantly in the cytoplasmic compartment [21]. To further understand the role of Clic1 in the hypothalamus, we conducted a series of experiments in an adult mouse hypothalamic cells line (mHypoA). These cells express Clic1 as well as the pro-feeding neuropeptides Npy and Agrp (Figure 1D). In a similar way observed in mouse hypothalamus, simulation of the fasted state, by nutrient depletion, resulted in increased expression of *Clic1*, as well as Npy and Agrp (Figure 1D). Importantly, IAA94 treatment blocked the fasting-induced increase in expression of *Npy*. In addition, IAA94 did not significantly reduce expression levels of Clic1 (Figure 1D) as IAA94 functionally inhibits Clic1 by preventing translocation to the transmembrane region [21,24]. Next, we determined if chronic obesity impacts Clic1 cellular localization, by studying hypothalamic samples from lean and obese mice. Chronic obesity resulted in an increase proportion of hypothalamic Clic1 localized to the membrane and lowered proportions in the cytoplasm compared with samples from lean mice (Figure 1E–G). Antibody specificity to Clic1 is confirmed by western blot (Supplemental Figure 1).

### 3.3 Clic1 KO mice eat less and weight less than WT littermates

**Figure 2:**
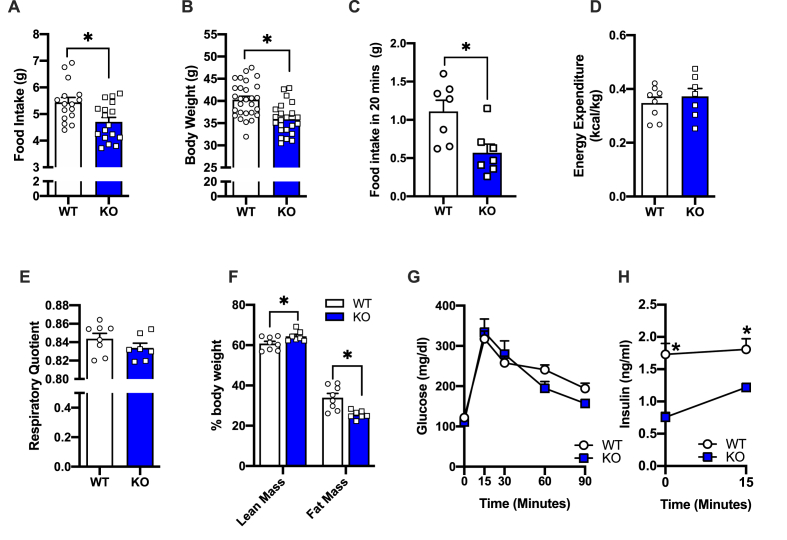
**Whole body knockout of *Clic1* is associated with lower food intake and body weight**. **A**. daily food intake (n = 7–8/group) **B.** Body weight (n = 7–8/group), **C.** Food novelty test, average daily consumption of 45% HFD when administered for 20 min per day (n = 7–8/group). **D.** Energy expenditure (n = 7–8/group), **F.** Respiratory quotient (n = 7–8/group), **F.** Average lean mass and fat mass (n = 7–8/group), **G.** Glucose tolerance (n = 7/group), **H.** Glucose stimulated insulin levels (n = 7/group). A–F ∗*p* < 0.05 Students t-test, G–H ∗*p* < 0.05, repeated measures ANOVA followed by Two-stage linear step-up procedure of Benjamini, Krieger and Yekutieli with 0.05 FDR.

We next studied the effect whole body Clic1 deletion on food intake, body weight and glucose homeostasis. Clic1 KO mice fed normal chow ate significantly less (Figure 2A) and weighed less (Figure 2B) than their WT littermates. In addition, when given access to highly palatable 45% HFD for 20 min per day, Clic1 KO mice showed less ‘motivation’ to overcome their innate fear of novelty and ate less HFD during this limited exposure time compared with their WT littermates (Figure 2C) suggesting Clic1 KO resulted in less motivation to consume calorically dense food. Clic1 KO mice were then placed in metabolic chambers to determine the impact of Clic1 ablation on energy homeostasis (Figure 2D and E). Clic1 KO mice displayed similar levels of energy expenditure (Figure 2D) and had no differences in respiratory quotient (Figure 2E) compared with WT littermates, suggesting the lower body weight was attributable to lower food intake. Clic1 KO mice also had increased lean mass and decreased fat mass, relative to their body weights (Figure 2F). Glucose tolerance tests in normal chow fed mice revealed similar levels of glucose tolerance (Figure 2G), with lower levels of insulin in KO mice, indicative of improved insulin sensitivity in the Clic1 KO compared with WT (Figure 2H).

### 3.4 Inhibition of food intake by IAA94 is specifically mediated through Clic1

**Figure 3:**
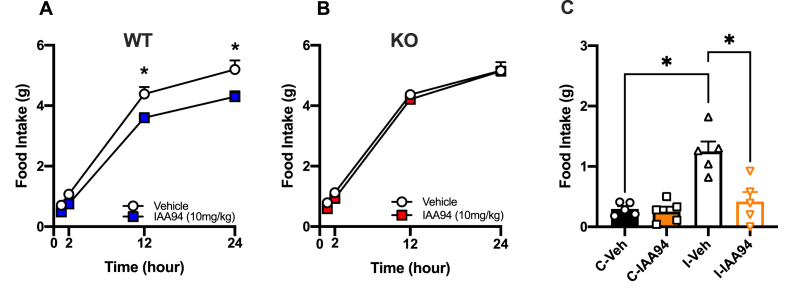
**IAA94 mediated reduction in food intake is mediated via Clic1**. Male (**A**) WT mice and (**B**) Clic1 KO were fasted for 23 h and then injected once with IAA94 (10 mg/kg) or vehicle (VEH) before being re-fed with normal chow and then food intake measured 24 h after re-feeding (n = 9–10/group). **C**. Food intake measured in WT mice during binge eating study (n = 5/group). WT mice were divided into one of 4 experimental groups; ‘Continuous’ (C-Veh or C-IAA94) and ‘intermittent’ (I-Veh or I-IAA94), ‘Continuous’ groups had *ad libitum* access to HFD throughout the study. The ‘intermittent’ groups received an initial 48-h acclimation to HFD followed by 5 days of access to NC only, before the HFD was then presented back to the mice for a short 2.5-h period and food intake measured. A–B ∗*p* < 0.05, repeated measures ANOVA, C. ∗*p* < 0.05, two-way ANOVA followed by Two-stage linear step-up procedure of Benjamini, Krieger and Yekutieli with 0.05 FDR.

To determine if pharmacological inhibition of Clic1 could affect food intake, we fasted mice and then treated with IAA94 before re-feeding. IAA94 has been widely used a potent inhibitor of Clic1 in many preclinical studies [[51], [52], [53]]. In fasted WT mice, IAA94 treatment significantly blunted 24hr food intake when mice were re-fed compared with vehicle treated controls. Importantly, when IAA94 was administered to Clic1 KO mice, there was no effect on fasting-induced re-feeding, proving that IAA94's effects on food intake are specifically mediated through Clic1 rather than other members of the Clic protein family (Figure 3A and B). To further explore the role of Clic1 in food intake in broader translation models of eating disorders, we tested whether IAA94 treatment impacted binge-eating in mice [30]. When mice were given intermittent access to HFD, they consumed significantly more of the palatable HFD than when it was continuously available. However, when IAA94 is administered prior to intermittent HFD exposure, this significantly lowered the amount of food that was consumed during this ‘binge eating’ period (Figure 3C).

### 3.5 Clic1 inhibition has a dose-dependent effect on reducing body weight and has an additional effect on weight loss when given in combination with GLP-1 agonist, Liraglutide

**Figure 4:**
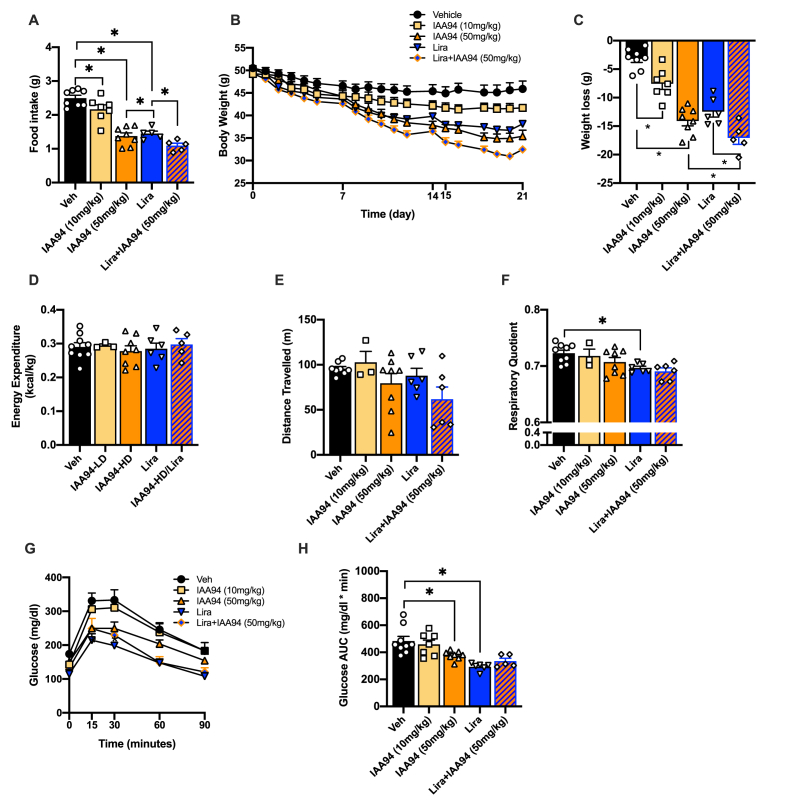
**Clic1 inhibition has a dose dependent effect on reducing body weight and has an additional effect on weight loss when given in combination with GLP-1 agonist, Liraglutide**. **A.** average daily food intake, **B.** weight loss, **C.** % weight loss, **D.** Energy expenditure, **E.** Activity, **F.** Respiratory Quotient, **G.** Glucose tolerance test, **H.** Area under the curve of GTT of HFD-fed obese mice treated with Vehicle (n = 9), IAA94 50 mg/kg (n = 8), IAA94 10 mg/kg (n = 7), Lira (n = 6), combo [IAA94 50 mg/kg + Lira] (n = 6) for 21 days. Metabolic chamber analysis was conducted during days 8–15 of treatment. A, C–F, H ∗*p* < 0.05 One-way ANOVA followed by Two-stage linear step-up procedure of Benjamini, Krieger and Yekutieli with 0.05 FDR. B, G ∗*p* < 0.05, repeated measures ANOVA.

To test whether Clic1 inhibition could reduce food intake and induce weight loss in the context of obesity, we administered IAA94 to diet-induced obese mice. Chronic treatment with IAA94 resulted in a dose-dependent reduction in food intake (Figure 4A) and body weight (Figure 4B and C). IAA94-induced reduction in body weight was attributed to a significant reduction in fat mass with no significant changes in lean mass in all groups, compared with vehicle treatment (Supplemental Figure 2A and B). IAA94 treatment at a dose of 50 mg/kg resulted in slightly greater weight loss than liraglutide, and importantly combination treatment of Liraglutide and IAA94 (50 mg/kg) induced greater weight loss than either drug alone (Figure 4B and C). No significant differences in energy expenditure (Figure 4D) or activity (Figure 4E) were observed, further suggesting Clic1 inhibition drives weight loss due by reducing food intake. IAA94 treatment did not impact respiratory quotient while liraglutide treatment resulted in slightly lower respiratory quotient levels indicative of a mild increase in fat oxidation rather than utilization of carbohydrate as a fuel source. IAA94 (50 mg/kg) alone or in combination with Liraglutide also significantly improved glucose tolerance compared with vehicle (Figure 1G and H) as a likely secondary effect to the lower body weight (Figure 1B and C). Importantly, IAA94 did not change Alanine transaminase (ALT) enzymatic activity, which is a marker of hepatocyte injury and toxicity, and in fact, IAA94 resulted in a trend in lower levels of ALT (Supplemental Figure 2C).

### 3.6 Clic1 inhibition reduces hyperphagia and weight gain in mouse model of Prader–Willi syndrome

**Figure 5:**
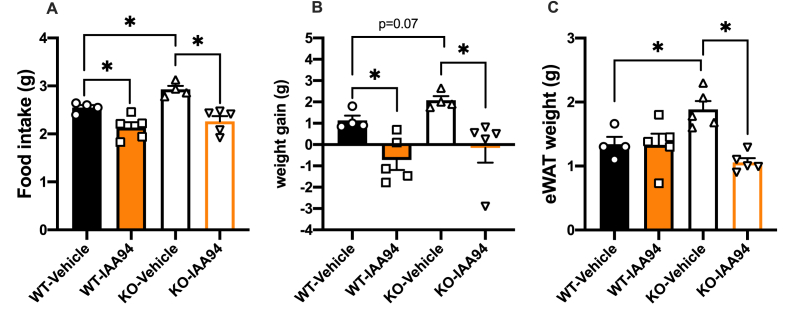
**Magel2 KO mice**. **A**. Average daily food intake, **B**. weight change and **C**. epididymal white adipose tissue weight of Magel2 WT and null mice treated with either Vehicle or IAA94 (10 mg/kg), n = 4–5 per group. ∗*p* < 0.05 Two-way ANOVA followed by Two-stage linear step-up procedure of Benjamini, Krieger and Yekutieli with 0.05 FDR.

To explore the broader potential of Clic1 inhibition in genetic models of obesity, we tested the effect of IAA94 in the Magel2 KO mouse model of Prader–Willi syndrome [54]. As previously described, HFD feeding of Magel2 KO mice resulted in hyperphagia and weight gain [54] (Figure 5A and B). Magel2 KO mice were fed HFD at 18 weeks of age and treated with IAA94 (10 mg/kg) or vehicle. Treatment with IAA94 significantly reduced food intake (Figure 5A) and prevented body weight gain in Magel2 KO mice (Figure 5B, Supplemental Figure 3). In addition, IAA94 treatment resulted in lower epididymal fat mass compared with vehicle treated control mice (Figure 5C).

### 3.7 Clic1 ablation had no effect on behavioral tests associated with depression or anxiety

**Figure 6:**
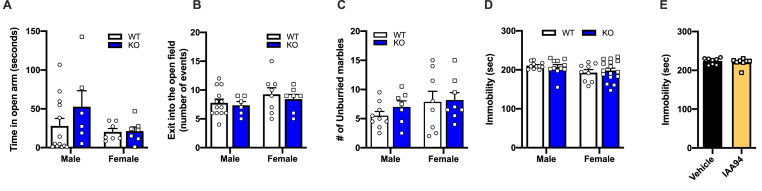
**Clic1 ablation had no effect on behavioral tests associated with depression or anxiety**. **A.** Time spent in open arm in elevated plus maze, **B.** Number of entries to closed arms during elevated plus maze test, (n = 6–13/group) **C.** Number of unburied marbles in marble burying anxiety test (n = 8–9/group). **D**. Time spent immobile in forced swim test in KO and WT mice (n = 9–16/group). **E**. Time spent immobile in forced Swim test in IAA94 (10 mg/kg) and Vehicle treated WT mice, (n = 7–9 per group). ∗*p* < 0.05 two-way ANOVA followed by Two-stage linear step-up procedure of Benjamini, Krieger and Yekutieli with 0.05 FDR.

Many previous efforts to develop centrally acting anti-obesity therapeutics have failed due to neuropsychiatric adverse effects including anxiety and depression [55]. Therefore, we tested whether ablation of Clic1 had any overt effects on depression or anxiety-like behavior in mice. Clic1 KO mice performed in a similar way to WT mice in all three behavioral assays, elevated plus maze (Figure 6A and B), marble burying assays (Figure 6C) and forced-swim tests (Figure 6D), suggesting Clic1 ablation had no overt effect on depression, compulsive behavior and anxiety. In addition, we treated a subgroup of mice with IAA94 and further confirmed that Clic1 inhibition had no impact on the forced swim test (Figure 6E), confirming that both genetic ablation and pharmacological inhibition of Clic1 had no measurable impact on behavioral tests associated with depression and anxiety in mice.

### 3.8 Clic1 ablation in the hypothalamus

**Figure 7:**
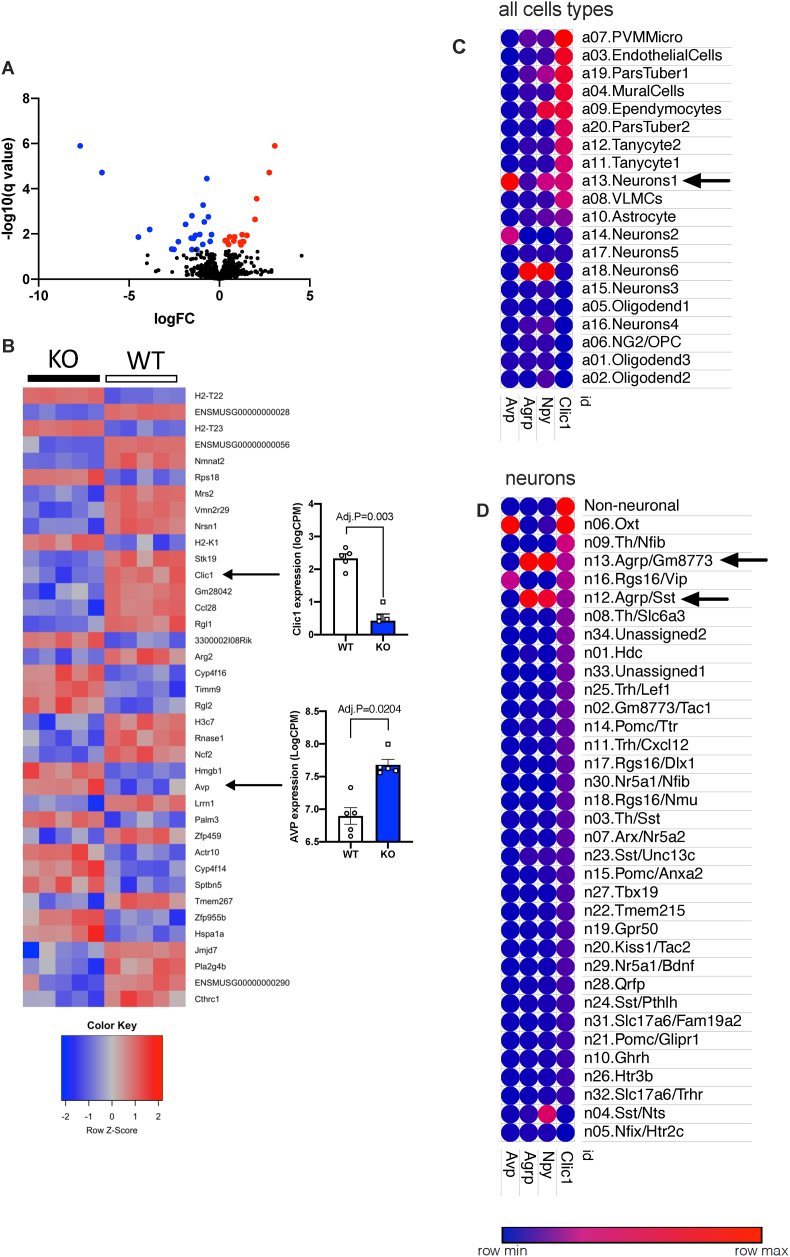
Hypothalamic RNA seq in Clic1 KO and WT mice. **A**. Volcano plot and **B**. Heat map of differentially expressed genes between the hypothalamus of Clic1 WT and KO mice. **C**. All cell-types and **D**. Specific neuronal population co-expression of Clic1 hypothalamic DEGs.

To explore potential hypothalamic molecular mechanisms underlying Clic1 regulation of energy balance, we conducted RNA sequencing from the mouse hypothalamus of normal chow fed WT and Clic1 KO mice (GSE229799). Differential gene expression analysis revealed 38 significantly differentially expressed genes (Adjusted *P* < 0.05), notably including *Clic1* (serving as an internal control) (Figure 7A and B, and Supplemental Tables 1 and 2). These differentially expressed genes represent potential molecular targets downstream of Clic1 that could also be therapeutic targets for modulation of food intake in obesity.

The RNA seq gene signature include Neurensin 1 (*Nrsn1*) and Leucine rich repeat neuronal 1 *(Lrrn1)* in which polymorphisms in these gene have been linked to changes in food intake [56] and obesity [57]. In addition, we noted significantly higher expression of key appetite regulating hormone Arginine vasopressin (*Avp*) (Figure 7B). Avp is robustly expressed in regions of the hypothalamus that regulated food intake, including the paraventricular hypothalamus (PVH) [58,59] and ARC [60]. Activation of Avp neurons in rats results in reduction in food intake [58] and injection of AVP dose dependently decreases food intake [61]. This association of lower *Clic1* and higher *Avp* suggests Avp may be a key component of Clic1 mediated lowering of food intake.

To determine which hypothalamic neurons express *Clic1*, we analyzed single-cell transcriptomic data (GSE93374) [60]. Clic1 is broadly expressed in many cell types but of particular interest is found in a neuronal population (a.13.Neurons.1) that also co-express Npy and Avp (Figure 7C). In addition, *Clic1* is broadly expressed in many non-neuronal cell types (Figure 7C) including perivascular macrophages and microglia (PVMMicro), endothelial cells, mural cells (form part of the blood brain barrier), ependymocytes (glial cell), tanycytes (glial cells that are part of the blood–brain barrier), and vascular and leptomeningeal cells (VLMCS).

We then examined the co-expression of *Clic1, Agrp, Npy* and *Avp* across 34 clusters of molecularly distinct neuronal subtypes (Figure 7D). This analysis confirmed that *Clic1* is found in *Agrp/Npy* expressing neurons (n13.Agrp/Gm8773 as well as n.12 Agrp/Sst). Additionally, *Clic1* is highly expressed in Oxytocin neurons (n.06.Oxt) that also express high levels of *Avp*. These hypothalamic single cell studies confirm that *Clic1* is expressed in neuronal populations that co-express neuropeptides with key roles in appetite regulation, including *Agrp, Npy* and *Avp*.

## 4 Discussion

In these studies, we describe the novel role of hypothalamic expression of *Clic1* in food intake regulation, specifically, overconsumption. Fasting drives an increase in *Clic1* expression in Agrp/Npy neurons. Genetic ablation of *Clic1* in mice results in a significant reduction in food intake and lower body weight compared with WT littermates. Furthermore, pharmacological inhibition of Clic1 results in suppression of food intake and promotes highly efficacious weight loss in obese mice. While IAA94 resulted in 28% reduction in body weight, combination treatment with both liraglutide and IAA94 resulted in an average of 35% weight loss, suggesting improved potential efficacy than many currently available anti-obesity drugs [62]. Clic1 inhibition was also highly efficacious in broader models of food intake including a mouse model of binge eating. This binge eating assay revealed that pretreatment with Clic1 inhibitor prevents the overconsumption of HFD when presented with this highly palatable food for a short period of time. Further detailed studies are needed to investigate the impact of Clic1 inhibition of food motivation and reward. IAA94 treatment also reduced food intake and weight gain in a Magel2 KO mouse model of the genetic condition Prader–Willi syndrome, suggesting additional areas of future clinical benefits of Clic1 inhibition. Many previous efforts to develop centrally acting anti-obesity therapeutics have failed due to neuropsychiatric adverse effects including anxiety and depression [55]. With a view to future translational potential, we studied the effect of Clic1 ablation on behavioral assays and confirmed that Clic1 ablation has no effect on depression or anxiety-like phenotypes in mice. Many of the previously described anti-obesity drugs modulate a number of neurochemical systems (dopamine, serotonin, noradrenaline, endocannabinoid) involved in regulation of mood, cognition and sleep and thus have an elevated risk for widespread neuropsychiatric adverse effects. Therefore, targeting specific regulators of overconsumption, and not targeting fundamental neurochemical systems is likely to reduce the risk of unwanted side effects.

Obesity often develops in response to small changes in food intake over time, and thus, is hard to delineate the underlying mechanisms. The neural circuits that regulate food overconsumption are highly complex and many molecular components of these circuits remain unknown [63]. Utilizing a model of drug-induced hyperphagia, that is applicable to both rodents and humans, enabled us to identify a novel role for Clic1 in the regulation of food intake. Clic1 is a particularly unusual protein as it exists in two forms: a soluble enzymatic form and a membrane-associated ion channel form with a single transmembrane region [14]. Transformation from the predominant glutathione-S transferase (GST)-like structure soluble form [15] to that of an integral membrane protein [16] that is triggered by changes in cellular pH and oxidative stress [17]. Clic1 inhibitor IAA94 prevents the translocation of Clic1 to the membrane-associated ion channel form [21,24]. Chloride ions play an important role in controlling neuronal excitability whereby accumulation of intracellular chloride drives hyperpolarization which leads to neuronal inactivation [64]. However, the strength of inhibition depends on the chloride ion gradient across the membrane. Therefore, increasing intracellular chloride could also decrease the concentration-driven entry of extracellular chloride which can lead to reduced inhibitory postsynaptic potential generated by chloride-permeable GABA receptors [64,65]. Agrp/Npy neurons receive inhibitory GABA inputs from leptin-responsive neurons [66]. Thus, it is possible that in the obese state, elevated levels of the membrane form of Clic1 results in increased concentration of intracellular chloride leading to reduced-inhibition of Agrp/Npy neurons which then drives feeding. Further studies are warranted to investigate the role of Clic1 specifically in Npy/Agrp expressing neurons. Moreover, chronic obesity results in a significant change in subcellular localization of Clic1 with an increased ratio of Clic1 in the membrane in the obese state [67]. It is also likely that the chronic inflammatory state associated with obesity plays a role in stimulating the persistent increase in membranal localization of Clic1. These observations provide a novel therapeutic strategy to block Clic1 translocation as a potential mechanism to reduce food intake and lower body weight.

## 5 Conclusion

These studies identify a novel role for Clic1 in the regulation of food intake and preclinical studies support the further development of Clic1 inhibitors for the treatment of obesity.

## Author contributions

OO, RCZ and MP conceptualized the studies. RCZ, DZ, AL, OO, BSC and MLV performed experiments and analyzed data. DP and HS provided the Clic1 KO mouse model. CAN and DRCF performed RNAseq analysis. OO and RCZ wrote and edited the manuscript.

## Data Availability

Data will be made available on request.

## Appendix A Supplementary data

Supplemental Figure 1 Clic1 KO mice. **A)** Western blot showing Clic1 antibody specificity, Immunoblots of spleen and hypothalamus in WT and Clic1 KO mice. **B)** Body weights of Clic1 KO mice (N = 7–8 per group). Supplemental Figure 1

Supplemental Figure 2 (**A**) Fat mass (**B**) Lean mass, (**C**) ALT levels after chronic treatment with IAA94. (Veh n = 9, IAA94 50 mg/kg n = 8, IAA94 10 mg/kg n = 3, Lira n = 6, combo [IAA94 50 mg/kg + Lira] n = 6). ∗*p* < 0.05 One-way ANOVA followed by Two-stage linear step-up procedure of Benjamini, Krieger and Yekutieli with 0.05 FDR. Supplemental Figure 2

Supplemental Figure 3 Body weight of Magel2 KO mice pre and post IAA94 treatment for 9 days. Supplemental Figure 3

Supplemental Table 1 Clic1 KO and WT hypothalamic RNAseq data normalized gene expression. **Supplemental Table 2**. Hypothalamic differentially expressed genes between KO v WT determined by RNA sequencing. Supplemental Table 1
