# Supplementary figures and images for: Targeting Clic1 for the treatment of obesity: A novel therapeutic strategy to reduce food intake and body weight
Source: Mol Metab. 2023 Aug 20;76:101794. doi: 10.1016/j.molmet.2023.101794 (PMC10480059; doi:10.1016/j.molmet.2023.101794)

**A**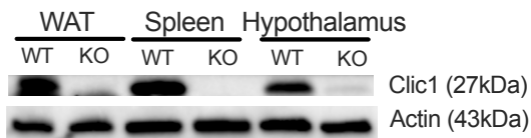**B**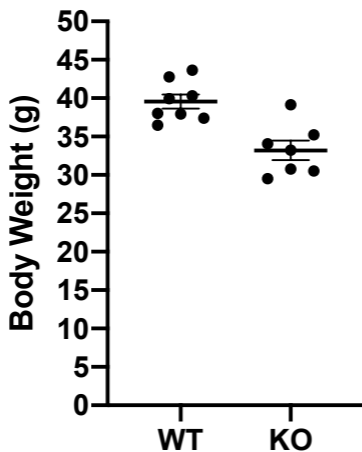

Supplement: Supplemental Figure 1 — Clic1 KO mice. A) Western blot showing Clic1 antibody specificity, Immunoblots of spleen and hypothalamus in WT and Clic1 KO mice. B) Body weights of Clic1 KO mice (N = 7–8 per group). [file mmc1.pdf]

**A**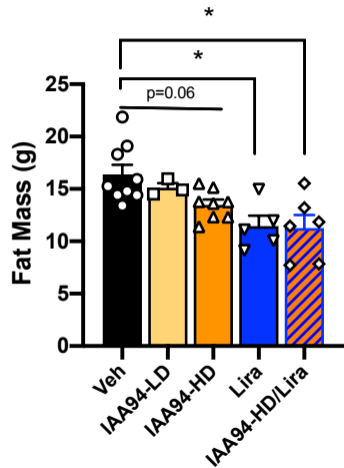**B**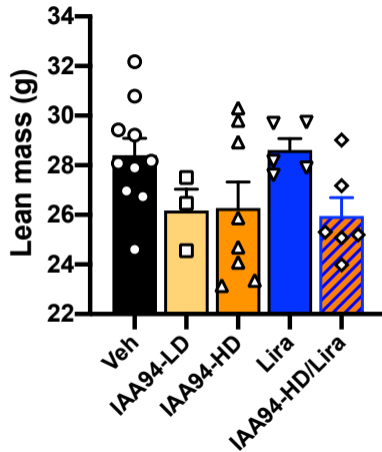**C**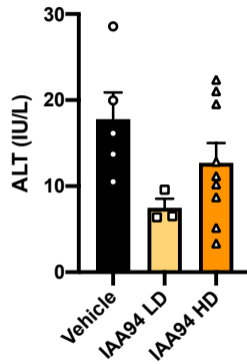

Supplement: Supplemental Figure 2 — (A) Fat mass (B) Lean mass, (C) ALT levels after chronic treatment with IAA94. (Veh n = 9, IAA94 50 mg/kg n = 8, IAA94 10 mg/kg n = 3, Lira n = 6, combo [IAA94 50 mg/kg + Lira] n = 6). ∗p < 0.05 One-way ANOVA followed by Two-stage linear step-up procedure of Benjamini, Krieger and Yekutieli with 0.05 FDR. [file mmc2.pdf]

**A**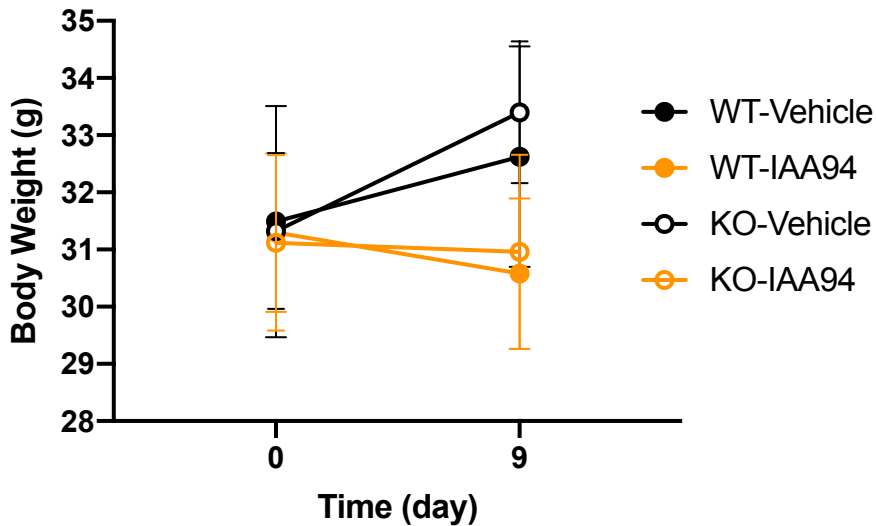

Supplement: Supplemental Figure 3 — Body weight of Magel2 KO mice pre and post IAA94 treatment for 9 days. [file mmc3.pdf]
